# One-carbon pathway and cognitive skills in children with Down syndrome

**DOI:** 10.1038/s41598-021-83379-7

**Published:** 2021-02-19

**Authors:** Francesca Antonaros, Silvia Lanfranchi, Chiara Locatelli, Anna Martelli, Giulia Olivucci, Elena Cicchini, Ludovica Carosi Diatricch, Elisa Mannini, Beatrice Vione, Agnese Feliciello, Giuseppe Ramacieri, Sara Onnivello, Renzo Vianello, Lorenza Vitale, Maria Chiara Pelleri, Pierluigi Strippoli, Guido Cocchi, Francesca Pulina, Allison Piovesan, Maria Caracausi

**Affiliations:** 1 Department of Experimental, Diagnostic and Specialty Medicine, (DIMES), Unit of Histology, Embryology and Applied Biology, University of Bologna, Via Belmeloro 8, 40126 Bologna, BO Italy; 2 Department of Developmental Psychology and Socialisation, University of Padova, Via Venezia 8, 35131 Padua, PD Italy; 3 Neonatology Unit, St. Orsola-Malpighi Polyclinic, Via Massarenti 9, 40138 Bologna, BO Italy; 4 Neonatology Unit, Department of Medical and Surgical Sciences (DIMEC), St. Orsola-Malpighi Polyclinic, University of Bologna, Via Massarenti 9, 40138 Bologna, BO Italy; 5 Medical Genetics Unit, Department of Medical and Surgical Sciences (DIMEC), St. Orsola-Malpighi Polyclinic, University of Bologna, Via Massarenti 9, 40138 Bologna, BO Italy

**Keywords:** Genetics, Neuroscience, Medical research

## Abstract

This work investigates the role of metabolite levels in the intellectual impairment of subjects with Down syndrome (DS). Homocysteine, folate, vitamin B12, uric acid (UA), creatinine levels and *MTHFR* C677T genotype were analyzed in 147 subjects with DS. For 77 subjects, metabolite levels were correlated with cognitive tests. Griffiths-III test was administered to 28 subjects (3.08–6.16 years) and WPPSI-III test was administered to 49 subjects (7.08–16.08 years). Significant correlations were found among some metabolite levels and between homocysteine levels and *MTHFR* C677T genotype. Moreover, homocysteine, UA and creatinine levels resulted increased with age. We did not find any correlation between metabolites and cognitive test score in the younger group. Homocysteine showed statistically significant correlation with WPPSI-III subtest scores when its level is ≥ 7.35 µmol/L, remaining correlated in higher thresholds only for non-verbal area scores. Vitamin B12 showed correlations with all WPPSI-III subtest scores when its level is < 442 pg/mL. The relevance of the present findings is the detection of a specific metabolite threshold related with a better or worse cognitive score, suggesting that vitamin B12 and homocysteine may have a role in cognitive development in children with DS.

## Introduction

Down syndrome (DS) [MIM: 190685] is caused by an extra copy of all^1^ or even a small part of^2^ human chromosome 21 (Hsa21) and it is the most common genetic cause of intellectual disability (ID). Hsa21 gene expression in cells and tissues from subjects with DS has been reported as altered^3^ and recently associated with changes in the metabolic profile that might be involved in the development of the clinical features^4^. In particular, there are descriptions of Krebs cycle substrate modification^5^, one-carbon metabolism perturbation^6^ and hydrogen sulfide (H_2_S) over-production caused by an over-expression of the cystathionine-beta-synthase (*CBS*) gene^7^. The *CBS* gene, encoded by Hsa21, is over-expressed in trisomy 21 cells^3^ and permanently removes homocysteine (Hcy) and serine to obtain cystathionine and, subsequently, cysteine catalyzing a trans-sulfuration reaction^8^. *CBS* might also have a role in dysregulated DNA methylation levels in subjects with DS, causing decreased Hcy availability and consequentially methionine (Met) synthesis^9^.

**Figure 1:**
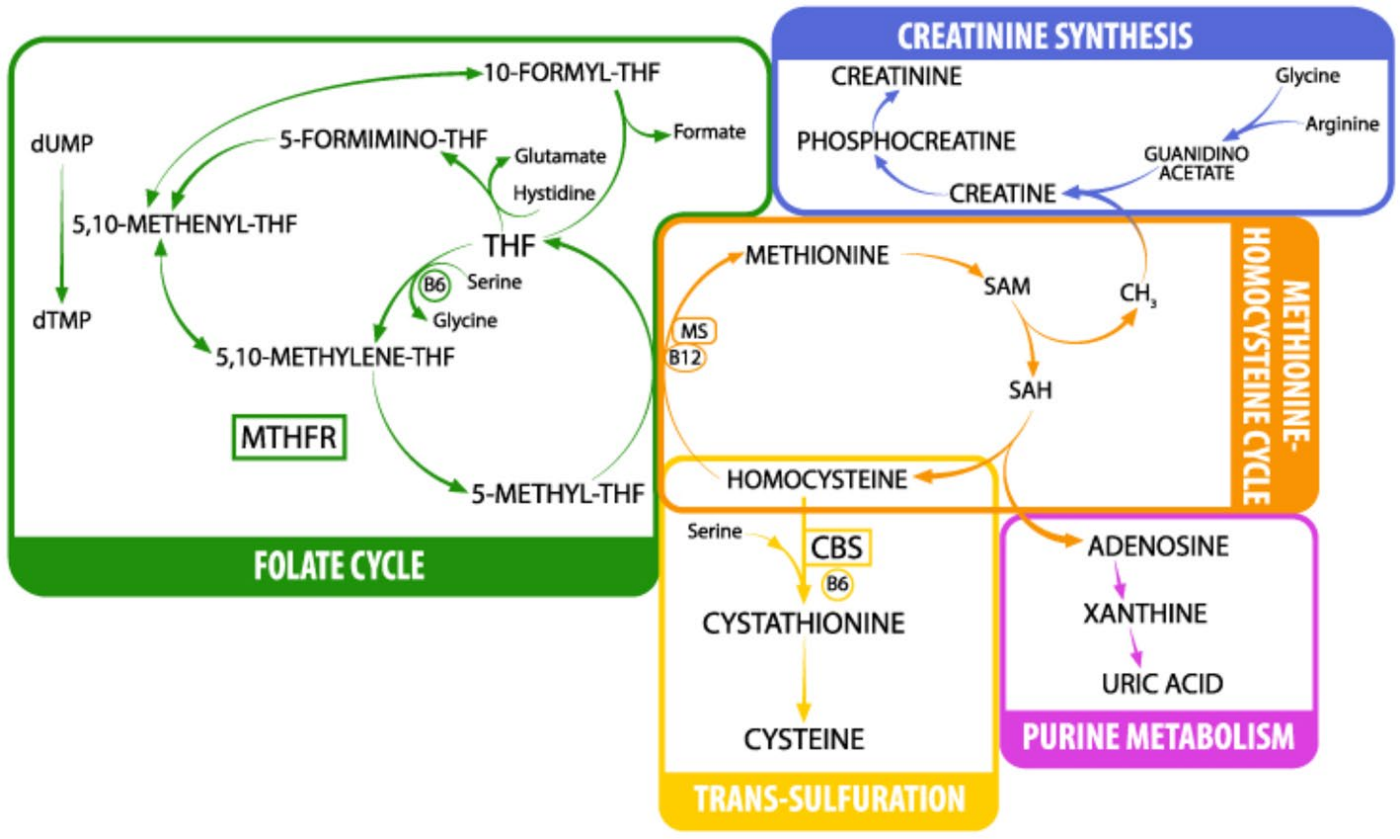
One-carbon metabolism. *THF* tetrahydrofolate, *dUMP* deoxyuridine monophosphate, *dTMP* deoxythymidine monophosphate, *MTHFR* methylene-tetrahydrofolate reductase, *MS* methionine synthase, *SAM* S-adenosylmethionine, *SAH* S-adenosylhomocysteine, *CBS* cystathionine beta-synthase. Image by Marcello Fedi.

Another critical enzyme for the regulation of Hcy concentration is 5,10-methylenetetrahydrofolate reductase (MTHFR) since it catalyzes the conversion of 5,10-methylenetetrahydrofolate (THF) to 5-methyl-THF. In the present work, we detected *MTHFR* C677T polymorphism (NG_013351.1:g.14783C>T; NM_005957.4:c.665C>T) due to its well-known correlation with increased Hcy plasma levels^10^. 5-methyl-THF is the major donor of the methyl group, together with trimethylglycine (betaine) for the conversion of Hcy to methionine (Met)^11^. Met can be transformed into S-adenosyl-methionine (SAM) that, together with arginine and glycine, can be used for the production of creatine (precursor of creatinine). SAM is one of the principal methyl donors and when it loses its methyl group becomes S-adenosyl-homocysteine (SAH). SAH can regenerate Hcy and adenosine with the latter being converted into uric acid (UA). Hcy and folate metabolism are both parts of the one-carbon cycle, and because of this, they are closely related. In addition, the conversion of Hcy to Met is catalyzed by 5-methyltetrahydrofolate-homocysteine methyltransferase (MS), a B12-dependent enzyme that uses (5-methyl-THF) the derivative of folate as a methyl donor (Fig. 1).

Hcy concentration has also been reported to be increased with low folate or vitamin B12 levels and lower with supplementation of vitamin B12 and folate^12^. Subjects with DS resulted to have a lower tolerance to the antifolate drug methotrexate (MTX) and their blood levels of vitamin B12 and folate have been reported to be lower than healthy controls^13^.

These results reinforce the idea that DS might be a metabolic disease due to the presence of biochemical alterations of fundamental pathways like the folate cycle, Hcy-Met cycle and Hcy trans-sulfuration^14^. Different studies show how Hcy and vitamin B12 could be involved in cognitive development. In a research study conducted in 2017, Murphy et al.^15^ noticed how an elevated maternal Hcy at preconception is associated with lower cognitive performance in children 4 months and 6 years after birth^15^ while vitamin B12 concentration was positively correlated with the cognitive score in 18 month old children^16^. ID is a constant feature in individuals with DS, even if it is present to an extremely variable degree^17^. Individual cognitive development is influenced by environmental factors^18–20^, but it may also involve biological mechanisms. Lejeune was the first who hypothesized that a “blocked” mechanism might determine the level of severity of ID^21^. Despite the individual variability, a specific cognitive profile of subjects with DS is reported in the literature: communication skills are generally found to pose the most significant challenges, non-verbal reasoning is a relative strength^22^, daily living skills are somewhat stronger, and socialization is the strongest adaptive skill^23–25^. The degree of ID changes over the lifespan of individuals with DS, in particular, their intelligence quotient (IQ) seems to decrease as they grow older due to their lower development rate with respect to typically developing children^26^. A previous study conducted in 2005 by Guéant and coll. on a cohort of 131 patients with trisomy 21 (mean age 20 years) reported how Hcy might influence cognitive development. They noticed how a lower IQ was significantly correlated with an Hcy level > 7.5 µmol/L (median) and an IQ < 40 was associated with Hcy concentration > 9.6 µmol/L (upper quartile)^27^.

This work investigates the correlation between *MTHFR* C677T polymorphism and Hcy plasma levels, the association among Hcy, folate, vitamin B12, UA and creatinine concentrations and their involvement in cognitive performance in subjects with DS.

## Results

A total of 147 children/young adults with a confirmed diagnosis of trisomy 21 and age > 2 years were studied (mean age = 11.49 ± 6.40; 60 females; 87 males). The information regarding age, sex, fasting state, the levels of Hcy, folate, vitamin B12, UA, creatinine, *MTHFR* C677T genotype and cognitive tests are listed in Supplementary Dataset S1. Cognitive test results were available for 77 DS children out of 147 subjects enrolled in the context of our clinical experimental study. Griffiths-III test was administered to 28 subjects (mean age = 4.55 ± 0.97) and WPPSI-III test was administered to 49 subjects (mean age = 11.34 ± 2.49).

### Correlation between MTHFR genotype and homocysteine plasma level

Hcy concentration level and *MTHFR* C677T polymorphism were studied in all 147 subjects enrolled. PCR–RFLP analysis (detailed in the Methods section) divided the 147 subjects into three genotype groups: 40 CC (27.2%), 69 CT (46.9%) and 38 TT (25.9%). These groups resulted in being homogeneous for sex after contingency test (p = 0.339) and for age after Kruskal–Wallis test (p = 0.718), so we searched for a correlation between Hcy concentrations and *MTHFR* genotype.

**Figure 2:**
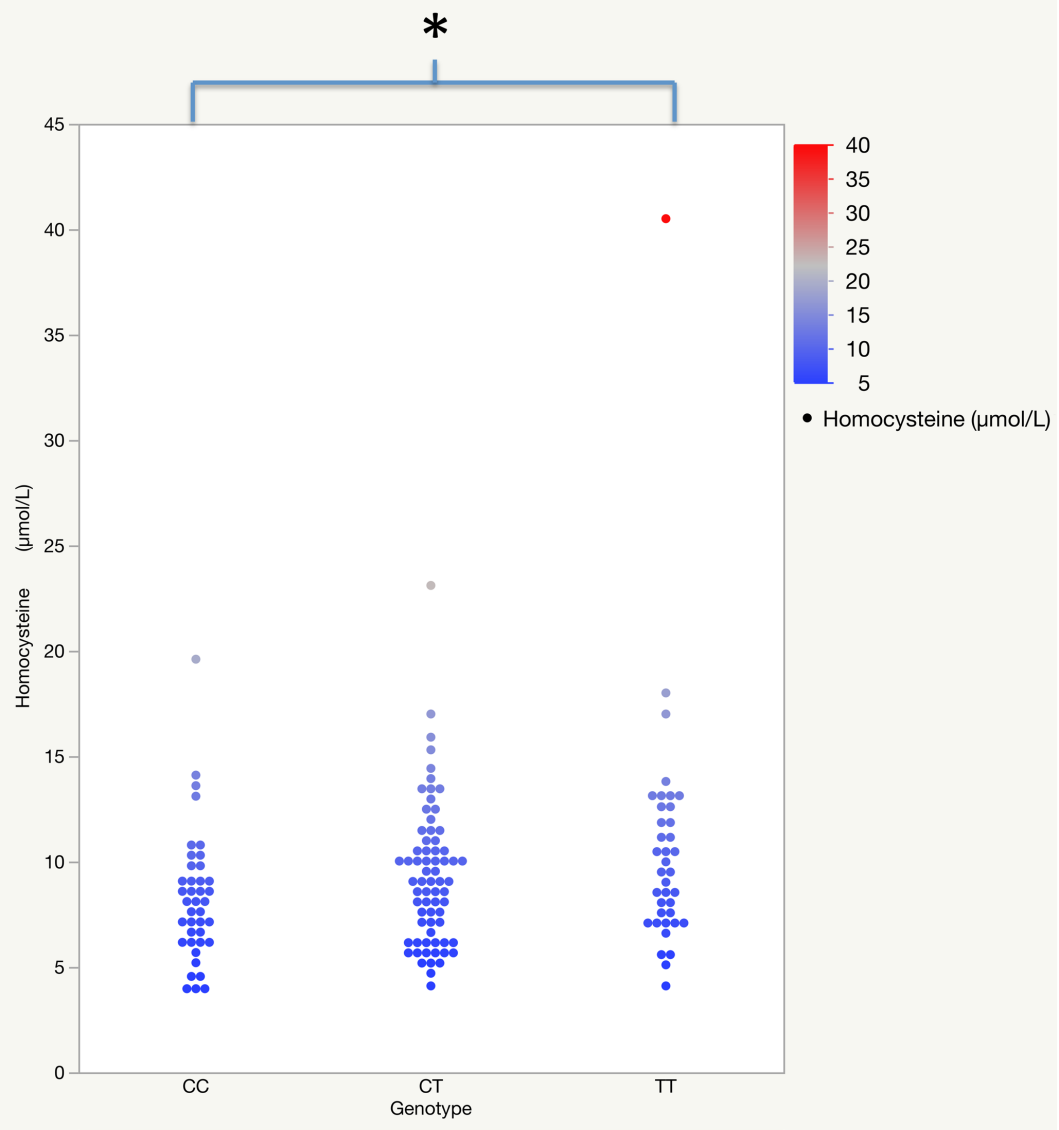
The variance of homocysteine level related to *MTHFR* genotype groups. One-way ANOVA analysis to compare Hcy concentrations among the three groups of *MTHFR* C677T genotype*.* The analysis includes outliers. *p-value =  0.035.

An unpaired t-test detected that Hcy concentration is independent of sex (p = 0.138) and fasting or non-fasting state (p = 0.234) while a significant linear correlation with age at blood draw was found (r = 0.467; p < 0.001) (Table 1). One-way analysis of variance (ANOVA) was performed to compare Hcy levels among the three groups of *MTHFR* C677T genotype showing a significant difference in Hcy mean concentration (µm/L) among the three groups (CC = 8.125; CT = 9.158; TT = 10.482) (p = 0.045). Post-hoc analysis with Tukey’s test showed a significant difference in Hcy mean concentration between TT and CC genotypes (p = 0.035) (Fig. 2). Scatter plots, reported as Fig. 2, clearly show a very significant outlier in the TT genotype. We then decided to calculate the p-value excluding strong outliers from each genotype group (outlier detection is detailed in the Methods section) due to their potential impact on the statistical significance. The statistical analysis highlighted DS125 (Hcy = 19.6 µmol/L) in CT genotype and DS009 (Hcy = 40.5 µmol/L) in TT genotype as strong outliers. Further one-way ANOVA still showed a significant difference in Hcy mean concentration (µm/L) among the three genotype groups (CC = 7.831; CT = 9.158; TT = 9.670) (p = 0.031). Post-hoc analysis with Tukey’s test confirmed a significant difference in Hcy mean concentration between TT and CC genotypes (p = 0.032).

**Table 1:** Linear correlations (r) between age and metabolites; unpaired t-test (t) between sex and metabolites; unpaired t-test (t) between fasting state and metabolites.

	Homocysteine		Vitamin B12		Folate		Uric acid		Creatinine	
Age	r	p	r	p	r	p	r	p	r	p
	0.467*	< 0.001*	− 0.253	0.002*	− 0.109	0.196	0.535*	< 0.001*	0.800**	< 0.001*
Sex (M/F)	t	p	t	p	t	p	t	p	t	p
	− 1.493	0.138	0.846	0.393	0.976	0.331	− 1.153	0.251	− 0.518	0.605
Fasting (yes/no)	t	p	t	p	t	p	t	p	t	p
	1.194	0.234	− 2.730	0.007*	− 1.190	0.236	2.227	0.028*	1.870	0.064

The analysis includes outliers. Further analysis after outlier exclusion is described in the text.

*p-value ≤ 0.05 or moderate correlation 0.4 < r < 0.7.

**Strong correlation with r ≥ 0.7.

### Correlation between metabolite concentrations and age, sex and fasting state

A linear correlation was established between the level of metabolites (Hcy, vitamin B12, UA and creatinine) and age at the time of blood sampling. In particular, we observed the increase of Hcy, UA and creatinine levels statistically correlated with the increase of age (Table 1). Vitamin B12 level was reported as statistically significant correlated with age but with a weak correlation strength. The unpaired t-test between sex and metabolite levels showed no statistically significant correlation, while the unpaired t-test between fasting state and metabolite levels highlighted that vitamin B12 and UA were affected by fasting state (Table 1).

### Correlations among metabolite levels

**Figure 3:**
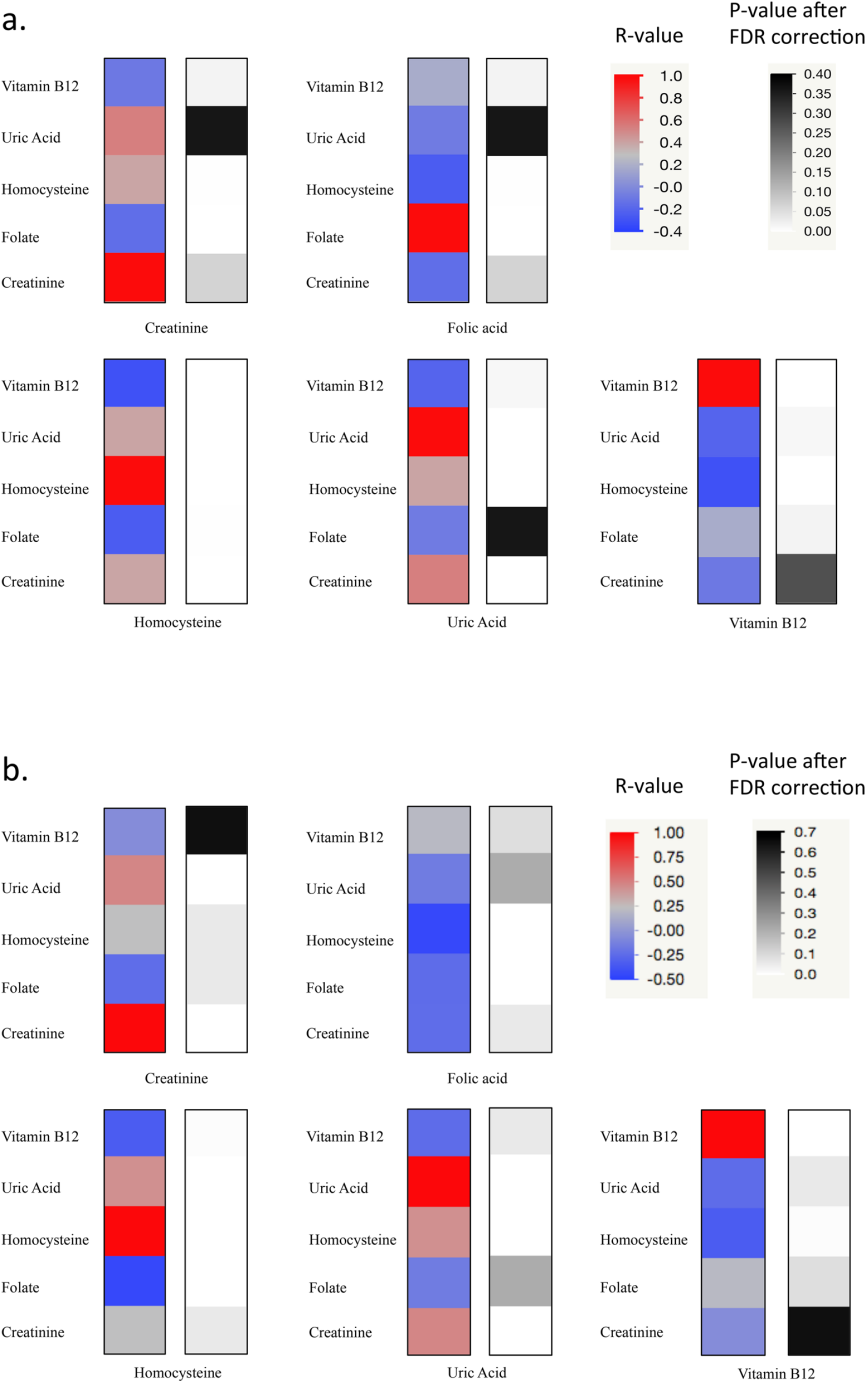
Heat Map of the metabolite level correlations among each other. (**a**) Partial correlation controlled by age at blood draw between the metabolite levels among each other. The analysis includes outliers. (**b**) Partial correlation controlled by age at blood draw between the metabolite levels among each other. The analysis excludes strong outliers.

Partial correlation controlled by age at blood draw between the level of each metabolite and the levels of all the other metabolites showed significantly moderate positive correlations: between Hcy and UA, (r = 0.456, p-value < 0.001 after False Discovery Rate (FDR) correction, see Methods section for details), between Hcy and creatinine (r = 0.417, p-value after FDR correction < 0.001) and between UA and creatinine (r = 0.541; p-value after FDR correction < 0.001) (Supplementary Table S1a, Fig. 3a). These analyses included only subjects in fasting state (n = 83).

Further analyses were assessed after removing strong outliers from fasting group. The statistical analysis reported two strong outliers (DS009, Hcy = 40.5 µmol/L; DS172, Hcy = 23.1 µmol/L) among the Hcy values (Hcy level ≥ 22.925 µmol/L) and two strong outliers (DS088, folate = 23.3 ng/mL; DS158, folate = 23.3 ng/mL) among the folate values (folate level ≥ 23.225 ng/mL). After the outlier removal, significantly moderate correlations were confirmed between Hcy and UA (r = 0.449, p-value after FDR correction < 0.001) and between UA and creatinine (r = 0.492; p-value after FDR correction < 0.001). Meanwhile, correlation strength between Hcy and creatinine decreased (r = 0.247, p-value after FDR correction 0.061) and a new negative moderate correlation emerged between Hcy and folate (r = -0.445, p-value after FDR correction < 0.001) (Supplementary Table S1b, Fig. 3b).

### Correlations between homocysteine, folate, vitamin B12, uric acid, creatinine levels and cognitive test scores

Being Hcy, folate and creatinine independent from fasting state, their concentration levels were tested for correlation with all subjects with Griffiths-III and WPPSI-III test scores (Table 2). For vitamin B12 and UA, which resulted dependent on fasting state, we tested these correlations only for fasting subjects (Table 2).

**Table 2:** Correlation between metabolite levels and Griffiths-III or WPPSI-III test scores.

		Griffiths-III test			WPPSI-III test			
		A AE	B AE	IQ	Total AE	Verbal AE	Non-verbal AE	IQ
Hcy n = 28 (G) n = 49 (W)	r	0.092	0.257	0.174	− 0.346	− 0.329	− 0.204	− 0.376
	p-value	0.660	0.216	0.375	0.016*	0.022*	0.164	0.008*
Folate n = 26 (G) n = 49 (W)	r	0.347	0.099	0.370	0.104	0.158	− 0.049	0.112
	p-value	0.089	0.638	0.063	0.482	0.285	0.741	0.442
Uric acid n = 12 (G) n = 27 (W)	r	0.410*	0.575*	0.363	− 0.023	− 0.161	0.267	− 0.189
	p-value	0.239	0.082	0.247	0.912	0.433	0.187	0.346
Creatinine n = 28 (G) n = 49 (W)	r	0.023	0.194	0.028	0.061	0.007	0.132	− 0.135
	p-value	0.913	0.353	0.888	0.679	0.963	0.369	0.355
Vit. B12 n = 11 (G) n = 27 (W)	r	0.054	0.313	0.117	0.282	0.276	0.169	0.386
	p-value	0.881	0.349	0.733	0.163	0.172	0.409	0.046*

Partial correlations (r) correct by age was done between Griffiths-III or WPPSI-III subtest scores and metabolite levels; bivariate correlations (r) were done between IQ scores and metabolite levels. Correlation between vitamin B12 (Vit. B12)/UA levels and Griffiths-III or WPPSI-III test scores was analyzed only in fasting subjects. The analysis includes outliers. Further analysis after outlier exclusion is described in the text.

*AE* age equivalent, *A* evaluation of “foundations of learning”, *B* evaluation of “language and communication”, *IQ* intelligence quotient, *(G)* number of subjects with Griffiths-III test scores, *(W)* number of subjects with WPPSI-III test scores.

*p-value ≤ 0.05 or moderate correlation 0.4 < r < 0.7.

Hcy concentrations resulted statistically significantly associated with total age equivalent (AE), verbal AE and IQ of WPPSI-III test with a weak negative correlation (Table 2). Vitamin B12 levels were associated with IQ scores of WPSSI-III test with a weak positive statistically significant correlation (Table 2).

Strong outliers were detected in the Griffiths-III group (DS088, folate = 23.3 ng/mL; DS044, creatinine = 0.59 mg/dL) and WPSSI-III group (DS158, folate = 23.3 ng/mL). Removing the outliers, no modification in statistical significance was detected among metabolite-cognitive correlations.

Taking as a model the study of Guéant et al.^27^ for Hcy, folate, vitamin B12, UA and creatinine, we used the concentration values of 25th, 50th or 75th percentiles as references for dividing the subjects into two groups, one with a metabolite concentration value lower than the reference (Low or L group) and the other with a concentration value higher than or equal to the reference (High or H group) (Supplementary Tables S2, S3). For vitamin B12 and UA affected by fasting state, it was not possible to correlate metabolite levels with L group of 25th percentile threshold and H group of 75th percentile threshold of Griffiths-III test because the number of subjects included in the analyses was insufficient (subjects < 4) (Supplementary Table S2).

In the younger group (3.08–6.16 years), we found no statistically significant correlation (Supplementary Table S2).

**Figure 4:**
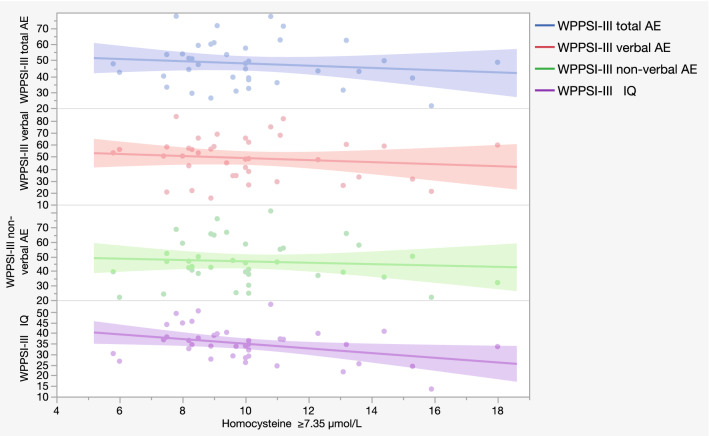
Scatter plot of the main correlation between homocysteine levels and WPPSI-III test scores. Correlation between homocysteine ≥ 7.35 µmol/L (H = High group of 25th percentile threshold) and WPPSI-III non-verbal AE scores, verbal AE scores, total AE scores and IQ scores of 37 subjects. Each dot represents a sample subject. Each line represents a correlation analyzed.

In the older group (7.08–16.08 years) the statistical analysis showed a moderate negative correlation between the H group of the 25th percentile value of Hcy (Hcy ≥ 7.35 µmol/L; 37 subjects) and the total AE scores (r =  − 0.455; p = 0.005), the non-verbal AE scores (r =  − 0.428; p = 0.009) and the IQ scores (r =  − 0.470; p = 0.003) (Supplementary Table S3, Fig. 4).

The non-verbal AE is the only area that still had a moderate negative correlation for Hcy ≥ 8.9 µmol/L, the H group of the 50th percentile threshold, (r =  − 0.457; p = 0.022; 26 subjects) and for Hcy ≥ 10.45 µmol/L, the H group of the 75th percentile, (r =  − 0.618; p = 0.043; 12 subjects) (Supplementary Table S3).

**Figure 5:**
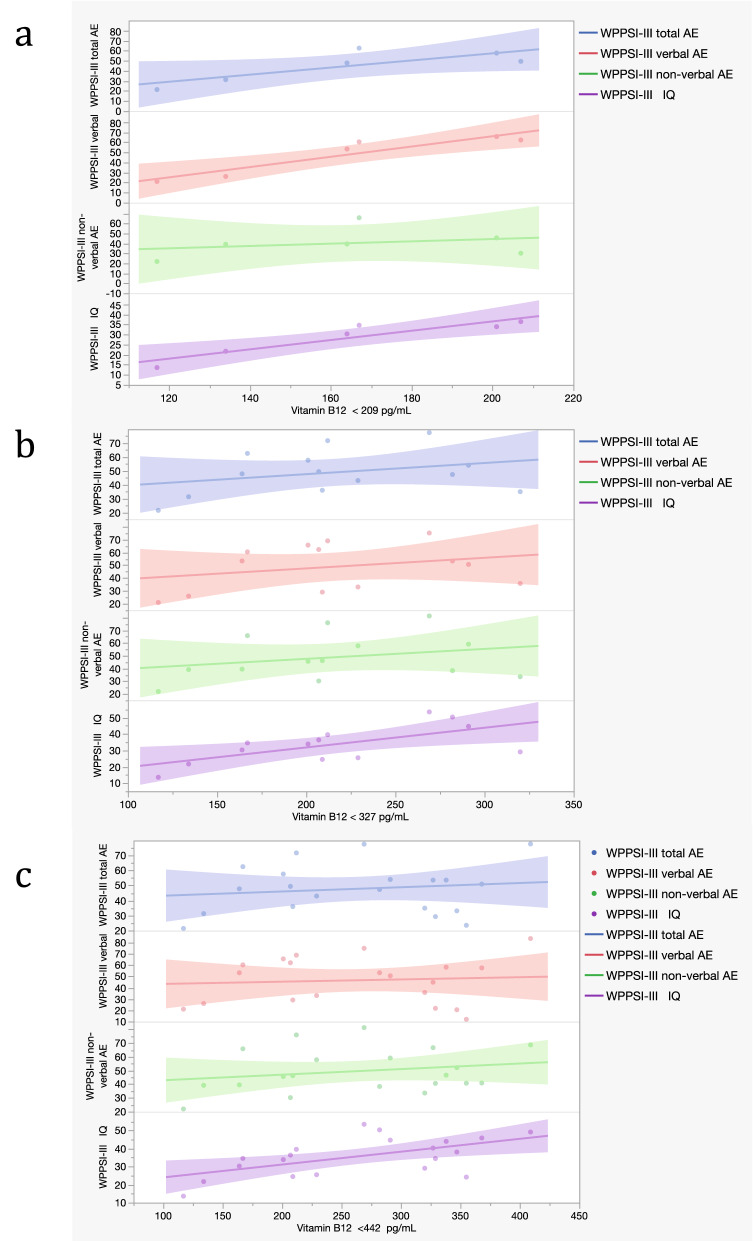
Scatter plot representing correlations between vitamin B12 and cognitive scores in the WPPSI-III group. (**a**) Correlations between vitamin B12 < 209 pg/mL (L group of 25th percentile threshold; 6 subjects) and WPPSI-III subtests scores. (**b**) Correlations between vitamin B12 < 327 pg/mL (L group of 50th percentile threshold; 13 subjects) and WPPSI-III subtests scores. (**c**) Correlations between vitamin B12 < 442 pg/mL (L group of 75th percentile threshold; 20 subjects) and WPPSI-III subtests scores (see Supplementary Table S3). Each dot represents a sample subject. Each line represents a correlation analyzed.

Among statistically significant correlations found for vitamin B12 percentile groups, vitamin B12 < 442 pg/mL (L group of the 75th percentile threshold; 20 subjects) showed a moderate positive correlation with verbal AE scores (r = 0.527; p = 0.020; 20 subjects), non-verbal AE scores (r = 0.606; p = 0.006; 20 subjects), total AE scores (r = 0.643 p = 0.003) and IQ scores (r = 0.572 p = 0.008) (Supplementary Table S3, Fig. 5).

After the exclusion of strong outliers (DS044, creatinine = 0.59 mg/dL and DS158, folate = 23.3 ng/mL in Griffiths-III group and DS088, folate = 23.3 ng/mL in WPSSI-III group) no significative modifications occurred in correlations performed between metabolites percetile groups and cognitive data (Supplementary Table S2b.2, S2e.2, S3b.2).

## Discussion

Many studies had already reported an impaired metabolic profile in subjects with DS^28,29^, so we decided to analyze Hcy, vitamin B12, folate, UA and creatinine levels due to their involvement in the one-carbon cycle.

First of all, our results confirmed a significant correlation between Hcy plasma levels and the *MTHFR* C677T genotype (Fig. 2), suggesting an association between the TT genotype with the increase of Hcy^10^. However, we cannot confirm this data because in our population, only 8 subjects present hyperHcy according to the range provided by “Laboratorio Unico Metropolitano” (LUM) (Supplementary Dataset S1).

Further analyses indicated a moderate positive correlation of Hcy, UA and creatinine concentrations among themselves (Fig. 3a, Supplementary Table S1a). A correlation of similar strength, between Hcy and UA, is still observed also excluding strong outliers from the analysis (Fig. 3b, Supplementary Table S1b). This condition could be explained by their involvement (directly or indirectly) in the one-carbon cycle. Vitamin B12 and folate concentrations are involved in Hcy level regulation^30^. Considering all metabolic data, we did not find any moderate or strong correlations among them in subjects with DS (Fig. 3a, Supplementary Table S1a). On the other hand, after the removal of strong outliers, a moderate negative correlation between Hcy and folate levels was found (Fig. 3b, Supplementary Table S1b). All metabolites analyzed resulted independent from sex and Hcy folate and creatinine were not affected by fasting state (Table 1).

In 50 of 77 subjects evaluated by Griffiths-III and WPPSI-III tests (see Methods section), several other metabolites were already analyzed with ^1^H-Nuclear Magnetic Resonance (H-NMR) in Antonaros et al.^31^, but none of them showed a correlation with IQ scores. These data suggest that metabolites analyzed in the present work could have a crucial role in cognitive function.

The first analysis performed in the older group including 49 subjects showed a weak negative significant correlation between Hcy levels and total AE (r =  − 0.346, p = 0.016), verbal AE (r =  − 0.329, p = 0.022) and IQ scores (r =  − 0.376, p = 0.008) (Table 2). Considering that the fasting state influenced vitamin B12 and UA, we decided to analyze only the fasting group involving 11 subjects for vitamin B12 and 12 for UA in the younger group (Griffiths-III test) and 27 subjects in the older group (WPSSI-III test).

Subsequently, we divided all the metabolite levels based on percentile threshold taking the study of Guèant et al.^27^ as a model (Supplementary Tables S2, S3).

These analyses showed, in the older group, that the verbal area had a weak strength of significant correlation with the Hcy levels only in the H group, (r =  − 0.332, p = 0.048, Hcy ≥ 7.35 µmol/L) and this is coherent with influences from different environmental variables (ex. family environment, school education, speech therapy, external stimuli, etc.) (Supplementary Table S3). The non-verbal area is the only one that conserved a stable and increased moderate and statistically significant correlation in all the H groups obtained with all three percentile thresholds, coherently with the idea that this area is less influenced by environmental factors^32^ and that the increase of Hcy concentration over 7.35 µmol/L might be already suggestive of a negative influence in this cognitive area in the DS population (similarly to what Guéant et al.^33^ found). The negative association of Hcy levels with cognitive scores confirmed literature data in which the increase of Hcy plasma levels is reported as an early marker of cognitive impairment in the elderly population^34^ and as increased in psychiatric phenotype^35^. The relevance and originality of the present findings is the detection of a specific metabolite threshold related with a better or worse cognitive score, independent from the metabolite physiological range. In this view, a control group was not necessary to validate our data because a reference interval of the investigated metabolites has also been reported in the literature^36,37^.

The vitamin B12 trend might indicate that, in the older children, a decrease of vitamin B12 levels under 442 pg/mL is correlated with a worse cognitive score and this threshold could be taken into account in the DS population which might have greater vitamin requirements (Supplementary Table S3). Vitamin B12 plays an important role in the synthesis of myelin, in the regeneration of nerves and in the antioxidant action of reduced glutathione^38^. For these reasons, a low level of vitamin B12 is associated with impaired neurotransmitter production and myelin lesion such as sclerosis of the spinal cord, polyneuritis, neuropathy, myelopathy, optic nerve atrophy and impaired cognitive functions^39^.

The analyses performed in the younger group might be influenced by a low statistical test power due to the small number of subjects included (Supplementary Table S2) but, on the other hand, the older group involved a larger number of subjects and this leads to a better interpretation of the correlations found.

In conclusion, we confirmed the previous literature regarding the association with *MTHFR* C677T genotype and Hcy levels and the correlation between metabolites like creatinine, Hcy and UA with age and among each other. Interesting data emerged from the analysis of correlations between cognitive scores and metabolite levels. In fact, an increasing level of Hcy above a specific threshold is associated with a decrease of cognitive test performance while a decreased concentration of vitamin B12 below a specific threshold is correlated with a worse cognitive test score. On the other hand, UA, folate and creatinine do not appear to be involved in cognitive development. Thanks to routine whole blood analyses, we managed to analyze these metabolites and their association with cognitive scores, but further studies including a larger number of subjects might lead to understanding the underlying mechanisms which cause impairment in cognitive development. Finally, specifically dedicated analyses should be performed in order to also investigate the epigenetic profile in DS subjects, considering that these subjects have hypermethylation of nuclear DNA^28^ and hypomethylation of mitochondrial DNA^40^ and methylation alterations were associated with cognitive functions^41^.

## Methods

### Blood processing, plasma preparation and metabolite detection

Information on drugs and vitamin supplements assumed by subjects with DS were collected at blood draw (Supplementary Dataset S1) and subjects whose samples might have been altered by these medications were excluded from the study.

Two aliquots of blood samples were collected at the Neonatology Unit of S. Orsola-Malpighi Hospital of Bologna in the context of the “Genotype–phenotype correlation in trisomy 21 (Down syndrome)” project (for ethical approval, see the corresponding paragraph below) and metabolite dosage was detected by LUM.

The first aliquot, used for *MTHFR* C677T genotyping and plasma Hcy dosage, was collected in ethylenediaminetetraacetic acid (EDTA)-coated blood collection tubes and plasma fraction was isolated within two hours from blood collection^42^. Plasma fraction was stored at − 80 °C until it was sent to LUM of Maggiore Hospital, Bologna, Italy for Hcy plasma level detection. The rate of NADH conversion to NAD+ (measured at A340 nm) is directly proportional to the concentration of homocysteine, and it was detected by an automated chemistry analyzer AU 400 Beckman Coulter (https://www.axis-shield.com/wp-content/uploads/2019/01/FHRWAU-FHRW100-200-100_Generic-HCY-IFU_EN_Ver201601.pdf).

The second aliquot was collected in EDTA free tubes and directly sent to LUM for routine blood analyses, which included folate, vitamin B12, UA and creatinine dosage. The quantitative determination of folate (https://www.beckmancoulter.com/download/file/phxB03898L-EN_US/B03898L?type=pdf) and vitamin B12 (https://www.beckmancoulter.com/download/file/phxA89094L-EN_US/A89094L?type=pdf) levels in human serum was obtained by chemiluminescent immunoassay by Beckman Coulter Immunoassay Systems. The quantitative determination of UA in human serum was detected by enzymatic colour test by Beckman Coulter analysers (https://www.beckmancoulter.com/download/file/phxBLOSR6X9813-EN_US/BLOSR6X9813?type=pdf). The quantitative determination of creatinine in human serum was detected by kinetic color test (Jaffé method) by Beckman Coulter AU analyzers. The compensated method traceable to the isotope dilution mass spectrometry (IDMS) reference method was used (https://www.beckmancoulter.com/download/file/phxBLOSR6X7817-EN_US/BLOSR6X7817?type=pdf).

The physiological range established by LUM were 0.5–1.2 mg/dL for creatinine, 2.6–7.2 mg/dL for UA, 3.1–19.3 ng/mL for folate, 145–914 pg/mL for vitamin B12 and 5–15 μmol/L for Hcy plasma level.

### Genotyping

*MTHFR* C677T polymorphism was analyzed according to our previously published protocol^43^ based on an improved polymerase chain reaction-restriction fragment length polymorphism (PCR–RFLP) reaction.

### Cognitive tests

Among the subjects enrolled who decided to participate in a cognitive evaluation, only the cognitive tests administrated within two months from blood sample collection were considered in order to correlate these data with metabolite levels. Thus, cognitive test results were available for 77 DS children out of the 147 subjects enrolled in the context of our clinical experimental study. Cognitive data of 50 subjects had already been shown in Antonaros et al.^31^ and, in this study, they are indicated with the same DS patient code.

Following a procedure increasingly more used in the field of ID and to have a more sensitive measure that avoided the floor effect, tests that were more appropriate for expected mental age rather than chronological were used to assess subject’s cognitive level^44^. Depending on the age of the participant, the Griffiths-III test^45^ was administered to 28 subjects aged from 3.08 to 6.16 years, while the WPPSI-III test^46^ was administered to 49 subjects from 7.08 to 16.08 years. Griffiths-III is a play-oriented developmental test. It provides an overall measure of a child’s development across different areas: “foundations of learning” (scale A), “language and communication” (scale B), “eye and hand coordination” (scale C), “personal-social-emotional abilities” (scale D) and “gross motor” (scale E).

For this study, scale A and scale B were considered. The former assesses critical aspects of learning during the early childhood years while the latter measures overall language development, including expressive language, receptive language, and, to a lesser extent, use of language to communicate socially with others (scale B). Raw scores were registered and transformed into AE scores which give us an idea of the level of development of any child in a given area, comparing that child with typically developing children of the same chronological age. The Wechsler Preschool and Primary Scale of Intelligence, third edition (WPPSI-III)^46^, consisting of different subtests, allows computation in three main indexes: verbal, non-verbal and total^19^. An IQ score was also calculated, by dividing the child's AE by his chronological age, multiplied by 100.

### Statistical analysis

Statistical analyses were carried out with SPSS Statistics (IBM, Version 25 for MacOSX). The p-value after FDR and Figs. 2, 3, 4 and 5 for this paper were generated using JMP Pro software, Version 14 of the SAS System for MacOSX. Copyright 2018 SAS Institute Inc. SAS and all other SAS Institute Inc. product or service names are registered trademarks or trademarks of SAS Institute Inc., Cary, NC, USA. If you did not perform the data analysis yourself or are not familiar with the specifics of the SAS software used for the data analysis, you should be able to obtain this information from either your institution’s SAS software administrator or the person who actually performed the data analysis. All continuous variables were reported as mean values ± SD from the mean. The statistical analysis was performed using the data available in Supplementary Dataset S1. For all results, a p < 0.05 was considered statistically significant. An r < 0.4 was considered as weakly correlated, 0.4 < r < 0.7 as moderately correlated and r > 0.7 as strongly correlated. The total of 147 subjects was divided into three groups depending on the *MTHFR* C677T genotype (CC, CT or TT).

Contingency tests were used to assess differences in sex among CC, CT and TT genotype groups, performed with SPSS Statistics software as follows: from the leading software Menu we selected “Analyze” and then “Descriptive statistics”, we then chose “Crosstabs”, we included Sex in “rows” and Genotype in “columns” boxes and finally selected “Chi-Square” in “Statistics” options. The Kruskal–Wallis test was used to assess differences in age distribution in Genotype groups (CC, CT, TT), performed with SPSS Statistics software as follows: from the leading software Menu we selected “Analyze” and then “Non parametric tests”, we then chose “Independent samples”, we included Age in “tests field” and Genotype in “groups” boxes and finally selected “Run”.

One-way ANOVA was used to assess differences in Hcy concentrations among the three genotypes. Analysis with the Tukey–Kramer method was performed as a post-hoc test for mean comparisons when ANOVA showed statistically significant results (p-value < 0.05). These analyses were performed with SPSS Statistics software as follows: from the leading software Menu we selected “Analyze” and then “General linear model”, we then chose “Univariate”; we included Hcy data in the “dependent variable” box and genotype data in “fixed factor” box; finally we went to “post hoc” options and chose “Tuckey test” in the “equal variances assumed” section.

The presence of outliers in metabolite level distribution was performed with SPSS Statistics software as follows: from the leading software Menu we selected “Analyze” and then “Descriptive statistics”, we then chose “Explore”, and included metabolite levels in “Dependent List”, finally in “Statistics section” we selected the “Outliers” and “Percentiles” options. SPSS Statistics software indicates strong outliers with an asterisk in the graph.

A linear correlation was used to determine if the correlation between age and metabolite levels existed. It was performed with SPSS Statistics software as follows: from the leading software Menu, we selected “Analyze” and then “Correlate”, we then chose “Bivariate correlation”, and finally we included our data in the “variables” box.

Correlations between sex and metabolite levels and between fasting/non-fasting state and metabolite levels were investigated with an unpaired t-test. One subject out of 147 without information about the fasting state was excluded from the statistical analysis. Unpaired t-test was performed with SPSS Statistics software as follows: from the leading software Menu we selected “Analyze” and then “Compare means”, we then chose “independent-samples T-test” and finally we included our data in the “test variables” box and inserted “Fasting/no fasting” in the “grouping variable” box.

Partial correlation corrected by age at blood draw was used to correlate each metabolite level with all other metabolite concentrations. Only fasting subjects were included. FDR correction was applied to p-values obtained from these statistical analyses. Partial correlation corrected by age was performed with SPSS Statistics software as follows: from the leading software Menu we selected “Analyze” and then “Correlation”, we then chose “Partial correlation” and finally we included our data in the main box and inserted “Age at blood draw” in the “checked by” box). To obtain p-value after FDR correction we created a file with all the p-values obtained from the previous analysis and, using JMP software, from the main menu we selected “Add-in”, then “False Discovery Rate p-value” command and finally inserted the p-value column in “p value column”.

Statistical analyses on cognitive tests were performed by dividing the subjects into two different groups according to the test used for collecting their cognitive data (Griffiths < 7 years; WPPSI > 7 years). Taking the study of Guéant et al.^27^ as a model, the threshold concentration of Hcy, folate, vitamin B12, UA and creatinine levels were calculated using quartiles performing the following steps using SPSS software: from the leading software Menu we selected “Analyze” and then “Descriptive statistics”, we then chose “frequencies”, and finally we included our data in the “variables” box and chose “quartiles” in the “statistics” option. The concentration value of the 25th, 50th or 75th percentiles was used as a reference for dividing the subjects into two groups, one with a metabolite concentration value lower than the reference (Low or L group) and the other with a concentration value higher than or equal to the reference (High or H group) (Supplementary Tables S2, S3). Partial correlation corrected by age at the time of cognitive test was performed to test correlations between percentile groups of Hcy, folate, vitamin B12, UA and creatinine levels and Griffiths-III “foundation of learning” and “language and communication” AE scores and total, verbal and non-verbal WPPSI-III AE scores. Pearson correlation was performed to test correlations between percentile groups of each metabolite and IQ score in Griffiths-III and WPPSI-III test.

### Ethics approval and consent to participate

The study was approved by the independent Ethics Committee of the University Hospital St. Orsola-Malpighi Polyclinic, Bologna, Italy (study number: 39/2013/U/Tess). Informed written consent was obtained from all participants or, if participants are under 18, from a parent and/or legal guardian. The patients, if over 18, or their legal guardians were requested and required to sign an informed consent form for the collection of blood and clinical data before taking part in the study. All procedures were carried out in accordance with the Ethical Principles for Medical Research Involving Human Subjects of the Helsinki Declaration.

## Supplementary Information

Supplementary Dataset S1.

Supplementary Table S1.

Supplementary Table S2.

Supplementary Table S3.

## Data Availability

The datasets generated and analyzed during the current study have been made available as Supplementary Dataset S1 (metabolite levels and cognitive data).
